# What Is the Influence of Morphological Knowledge in the Early Stages of Reading Acquisition Among Low SES Children? A Graphical Modeling Approach

**DOI:** 10.3389/fpsyg.2018.00547

**Published:** 2018-04-19

**Authors:** Pascale Colé, Eddy Cavalli, Lynne G. Duncan, Anne Theurel, Edouard Gentaz, Liliane Sprenger-Charolles, Abdessadek El-Ahmadi

**Affiliations:** ^1^Laboratoire de Psychologie Cognitive (UMR Centre National de la Recherche Scientifique 7290), Aix-Marseille Université, Marseille, France; ^2^Laboratoire d'Etude des Mécanismes Cognitifs (EA 3082), Université Lyon 2, Lyon, France; ^3^Psychology, University of Dundee, Dundee, United Kingdom; ^4^Laboratoire du Développement Sensori-Moteur, Affectif et Social (SMAS), University of Geneva, Geneva, Switzerland; ^5^Laboratoire de Neurosciences Sensorielles et Cognitives (UMR Centre National de la Recherche Scientifique 7260), Aix-Marseille Université, Marseille, France

**Keywords:** morphological awareness, vocabulary, phoneme awareness, reading acquisition, first-graders, low SES, graphical modeling, structural equation modeling

## Abstract

Children from low-SES families are known to show delays in aspects of language development which underpin reading acquisition such as vocabulary and listening comprehension. Research on the development of morphological skills in this group is scarce, and no studies exist in French. The present study investigated the involvement of morphological knowledge in the very early stages of reading acquisition (decoding), before reading comprehension can be reliably assessed. We assessed listening comprehension, receptive vocabulary, phoneme awareness, morphological awareness as well as decoding, word reading and non-verbal IQ in 703 French first-graders from low-SES families after 3 months of formal schooling (November). Awareness of derivational morphology was assessed using three oral tasks: Relationship Judgment (e.g., do these words belong to the same family or not? heat-heater … ham-hammer); Lexical Sentence Completion [e.g., Someone who runs is a …? (runner)]; and Non-lexical Sentence Completion [e.g., Someone who lums is a…? (lummer)]. The tasks differ on implicit/explicit demands and also tap different kinds of morphological knowledge. The Judgement task measures the phonological and semantic properties of the morphological relationship and the Sentence Completion tasks measure knowledge of morphological production rules. Data were processed using a graphical modeling approach which offers key information about how skills known to be involved in learning to read are organized in memory. This modeling approach was therefore useful in revealing a potential network which expresses the conditional dependence structure between skills, after which recursive structural equation modeling was applied to test specific hypotheses. Six main conclusions can be drawn from these analyses about low SES reading acquisition: (1) listening comprehension is at the heart of the reading acquisition process; (2) word reading depends directly on phonemic awareness and indirectly on listening comprehension; (3) decoding depends on word reading; (4) Morphological awareness and vocabulary have an indirect influence on word reading via both listening comprehension and phoneme awareness; (5) the components of morphological awareness assessed by our tasks have independent relationships with listening comprehension; and (6) neither phonemic nor morphological awareness influence vocabulary directly. The implications of these results with regard to early reading acquisition among low SES groups are discussed.

## Introduction

An association between reading achievement and socio-economic status (SES) has been reported consistently across decades of research (Duncan and Seymour, 2000; O'Connor et al., 2009; Cabell et al., 2013; Hemmerechts et al., 2017). The achievement gap between the most and least advantaged children is apparent at kindergarten entry and persists throughout the school years (Kieffer, 2013). Children from low-SES families are also known to show delays in aspects of language development which underpin reading acquisition such as vocabulary and listening comprehension (Scarborough, 2001; Muter et al., 2004), and lower levels of skill in oral language production and comprehension, extending from early childhood into high school and beyond (Hoff, 2006; Huttenlocher et al., 2010; Fernald et al., 2013). The aspect of language that appears most susceptible to social disadvantage is vocabulary size when investigated using maternal report, spontaneous speech and standardized tests in assessing expressive and receptive vocabulary (Hoff, 2012; Fernald et al., 2013). Standardized language tests also reveal discrepancies between higher and lower SES children when measures of grammatical development and complex syntax comprehension are included (Dollaghan et al., 1999; Huttenlocher et al., 2002).

The interaction between oral language and reading acquisition has recently been receiving much attention among children from low SES families (Tse and Nicholson, 2014). Two oral language skills have been the main focus of this research, namely, vocabulary knowledge and phoneme awareness (Hoff, 2012). In contrast, few studies have focused on how morphological awareness develops in low SES groups or on the relationship between morphological awareness and reading acquisition (Apel and Diehm, 2014); indeed, with French-speaking children, there have been no studies conducted on these issues.

Morphological awareness has been defined as the ability to identify and manipulate the smallest segments of meaning within words (Carlisle, 1995). This skill has been associated in the general population with vocabulary learning and with reading acquisition beyond the initial stages of schooling (Anglin, 1993; Deacon and Kirby, 2004; Kirby et al., 2012). The present study investigated the very *early* stages of reading acquisition among low SES children with the aim of examining how morphological knowledge relates to other oral language components such as listening comprehension, vocabulary, and phonemic awareness which are known to impact reading development (for a review, see Kirby et al., 2008).

### Predictors of first grade reading skills in typical populations

As kindergarten and first grade is the ideal point at which to intervene to reduce the risk for low SES children of later reading difficulties (Suggate, 2016), it is important to understand how early language and literacy typically interact during this period. According to the well-known simple view of reading (SVR, Gough and Tunmer, 1986), reading comprehension can be decomposed broadly into two components, word recognition (D), and oral language comprehension, both of which are necessary and equal in importance, but which, nevertheless, represent distinct abilities (see also Hoover and Gough, 1990; Tunmer and Chapman, 2012). Word recognition is defined as the ability to rapidly generate the phonological or orthographic codes from a printed word in isolation to allow identification. Measures of this component are assumed to be developmentally constrained (Hoover and Gough, 1990; Tunmer and Greaney, 2010). In the early stages of learning to read, measures of both decoding and visual word recognition are useful to assess the grapheme-phoneme conversion skills required for independent reading as well as the orthographic skills which allow recognition and generalization of information across familiar words. In the SVR model, oral language comprehension (originally linguistic comprehension) represents all of the verbal abilities involved in the understanding of an oral message such as words, sentences and discourse (Kirby and Savage, 2008). Although still a matter of debate, the most commonly used oral language comprehension tests assess vocabulary knowledge and oral sentence processing abilities, also labeled listening comprehension (see Keenan et al., 2008; Thompson et al., 2015).

The SVR model offers a useful general framework for researchers trying to identify the abilities involved in reading acquisition during first grade. The model does not aim to explain the development of visual word recognition but rather to show how this skill contributes together with oral language comprehension to reading comprehension development. In the present study, we were interested in knowing how visual word recognition develops with respect to oral language comprehension in the very early stages of reading acquisition (after only 3 months of formal instruction, in November of Grade 1). Some studies have questioned the assumption in the SVR that the visual word recognition and oral language components are independent (see Tunmer and Chapman, 2012; Wagner et al., 2015). For example, Ouellette and Beers (2010) assessed typically developing first-graders on measures of phonemic awareness (the ability to consciously analyze oral words into their sound constituents (phonemes), which proved to be the best predictor of reading success; Landerl et al., 2013), pseudoword reading (decoding skills), irregular word reading, listening comprehension, receptive vocabulary, and reading comprehension. Using regression techniques, they found that both decoding skills and vocabulary (depth) contributed significant variance to irregular word recognition. While this study did not use a confirmatory factor analysis (CFA) to test whether individual differences in word recognition and oral language comprehension skills show “unity” or “diversity” (e.g., Miyake et al., 2000), these results suggested that the indicators of these constructs are generally correlated. The nature of these constructs and their relations should be further explored.

A substantial body of research indicates that from Grade 2 onwards, morphological awareness also contributes to reading competence (word recognition and reading comprehension) independently of vocabulary, phonological awareness and orthographic processing (Casalis and Louis-Alexandre, 2000; Carlisle, 2003; Deacon and Kirby, 2004; Carlisle and Stone, 2005; Roman et al., 2009; Bowers et al., 2010; Kirby et al., 2012). However, for first graders, the evidence is contradictory. With English-speaking children, Carlisle and Nomanbhoy (1993) showed that morphological skills predict word reading performance independently of phonological skills; although the proportion of variance explained is smaller, 4 and 33.6%, respectively (see also Wolter et al., 2009; Apel and Lawrence, 2011; Deacon, 2012; Kruk and Bergman, 2013; and with Dutch-speaking first graders, Rispens et al., 2008). Nonetheless, Kirby et al. (2012) failed to find any evidence that morphological awareness contributes significantly to word reading until the third grade (see also Law and Ghesquière, 2017). With French-speaking children, the evidence is also contradictory since Sanchez et al. (2012) reported a significant contribution from morphological awareness to word reading in first grade but Casalis and Louis-Alexandre (2000) did not find a similar result until the second grade. However, all of these studies are difficult to compare since they used different tasks, both in assessing word recognition and morphological awareness. It is likely that this contributes to the inconsistency across results as Apel et al. (2013b) have shown that the particular skills measured by different morphological awareness tasks impact on their ability to predict word reading performance.

Among the rare studies conducted on morphological awareness and the development of word-reading, very few have explored the relationship between morphological skills, phonological awareness and vocabulary and *how these skills interact in shaping early reading development*. One reason for this is that these studies have tended to rely on hierarchical regression techniques, which are very helpful in identifying the contribution of one skill in predicting the development of reading independently of other skills but are much less informative when it comes to explaining *how different skills interact in a network of “causal” relations to shape this development*. For example, in order to isolate the distinct contribution of morphological awareness to word reading among first-graders, Carlisle and Nomanbhoy (1993), Apel and Lawrence (2011), Wolter et al. (2009), and Kirby et al. (2012) all controlled for phonological awareness, while Sanchez et al. (2012) controlled for vocabulary and only Rispens et al. (2008) controlled for both phoneme awareness and vocabulary. This set of results showed that there are aspects of phonemic and morphological awareness and vocabulary that make independent contributions to word reading but the findings say nothing about direct or indirect interrelationships among these variables in shaping word reading development. Our study aims to look more closely at these interrelationships.

### Predictors of first grade reading skills in low SES groups

By studying 394 children from low SES families, Gentaz et al. (2013) were able to use multiple regression analyses to show that, at the end of first grade, reading comprehension performance as assessed by reading short sentences was explained by listening comprehension as assessed by oral sentence comprehension (8.89%), decoding (33.99%), and vocabulary (5.45%) skills (see also Gentaz et al., 2015). Although, the influence of decoding was the most important predictor, the authors did not carry out regression analysis on these decoding skills. In contrast, Fluss et al. (2009) with 1,062 first graders from low SES Parisian families found that the variance in decoding as measured by pseudoword reading was almost entirely accounted for by phonemic awareness and rapid naming (27% of the variance).

To our knowledge, only one study by Apel et al. (2013b) has focused on the development of morphological skills and their influence on the emergence of word reading among first-graders from low SES families. In their study of 44 English-speaking first-graders, morphological awareness did not explain any additional unique variance over and above phonemic awareness for either word or pseudoword reading. In another study with a larger sample (*N* = 304) and more variety in socioeconomic status levels (although predominantly lower SES), Kim et al. (2013) found that phonological and morphological awareness and vocabulary were each unique predictors of first grade word reading. The origin of the discrepancy between these results is unclear and requires further exploration as it could be due to differences in the tests used to measure morphological awareness or else sample differences such as size or SES composition.

### The present research

Previous studies have reported contradictory results concerning the involvement of morphological awareness in the early phases of word reading among typically developing English- and French-speaking children. Even when studies have reported a clear effect of morphological awareness in word reading, there is no clear picture of the dependencies among morphological awareness, phoneme awareness and vocabulary. One reason for this might be that the majority of these studies used hierarchical regression analyses, involving only linear regression coefficients between a set of independent variables and a dependent variable.

An alternative approach yet to be conducted in this area would be to explore the co-variability among a large set of observed variables in terms of a smaller set of latent variables or factors. When applying this kind of reduction via exploratory or CFA, the assumption is made that an underlying causal model exists. A further possibility would be to combine CFA and regression analysis. This combination often invokes a measurement model that defines factors using observed variables (indicators) and a structural model that imputes directed relationships between factors. This combination is known as Structural Equation Modeling (SEM) (Bollen, 1989), and is generally used when there is a theoretical model to be tested by comparing its predictions with the data. With second graders at risk for reading difficulties, Nagy et al. (2003) used structural equation modeling to evaluate the contribution of phonological and morphological awareness, vocabulary, and orthographic processing to word reading. Oral vocabulary and orthographic processing contributed uniquely to word reading and morphological awareness contributed uniquely to reading comprehension. As morphological awareness and vocabulary were significantly correlated, the authors concluded that morphological awareness may contribute indirectly to word recognition via oral vocabulary. Recently, with third graders, Levesque et al. (2017), found that morphological awareness displayed both direct and indirect (via word reading) pathways to reading comprehension (phonological awareness and non-verbal ability were included in the model). There was no effect of vocabulary on word recognition and reading comprehension when morphological awareness was taken into account.

In these studies, the SEM approach was largely confirmatory rather than exploratory. However, in practice, the dichotomy “confirmatory” vs. “exploratory” should not be viewed in terms of which method to use. In fact, these approaches are complementary: exploratory data analysis searches for patterns of relationships while confirmatory data analysis makes use of statistical hypothesis testing on predicted models (Kiiveri and Speed, 1982; Bollen, 1989).

Regression, CFA and SEM require prior knowledge to completely specify a model but often there is insufficient prior knowledge to do that. Although still uncommon in studies of reading, Graphical Modeling is a data-driven approach for identifying and exploratory modeling the network structure based on a set of multivariate variables. It uses graph theory that enable concise representations of associations between variables. Graphical models provide a framework for modeling how these variables are mutually related and how conditional independence structures can be represented graphically. As Malave (2008) observes “the graphical Gaussian model (Dempster, 1972; Whittaker, 1990; Lauritzen, 1996; Edwards, 2000) models the data as multivariate Gaussian, but constrains the inverse of the covariance matrix to have a zero for all pairs of variables which are conditionally independent” (their correlations are zero given the rest of the variables). The inverse of the covariance matrix (called the precision matrix) is related to the partial correlation matrix. Edwards (2000) explains this as follows: “two variables are independent given the remaining variables if, and only if, the corresponding element of the inverse covariance is zero.” The graph of this model is formed by connecting two nodes with an edge if the corresponding partial correlations are not set to zero. Undirected relationships can also act as a starting point for further investigation with techniques such as SEM (see Kiiveri and Speed (1982) for a discussion of the relationship between partial correlation, graphical Gaussian models and SEM). Rosa et al. (2011) describe how “a recursive causal structure can be represented by a Directed Acyclic Graph (DAG), which is a set of variables (or nodes) connected by directed edges (arrows),” when they are not conditionally independent. Kiiveri and Speed (1982) introduced many examples of DAGs making several points concerning their parameterization, identification, estimation, fitting, and comparison. A DAG may be specified by three ways: prior knowledge incorporated in confirmatory approaches, guessing-and-testing, and discovery algorithms (Spirtes et al., 2001).

Numerous studies suggest that the early stages of word reading development depends crucially upon oral language skills. However, the majority of these studies focus on phonological skills, especially phonemic awareness as it appears to critically influence the development of reading skills (see for example, Hulme and Snowling, 2013). Little is known with regard to the exact influence of other oral language skills such as vocabulary, morphological awareness and listening comprehension and their mutual influence in the early stages of word reading and their relationship with phonemic awareness. In fact, some researchers suggest that oral language comprehension skills such as vocabulary, morphological awareness and listening comprehension may influence the development of reading comprehension (see Hulme and Snowling, 2013, for similar position). In order to show whether these skills can also contribute to the early acquisition of word reading, we assessed listening comprehension (comprehension of oral sentences), receptive vocabulary, phoneme awareness, morphological awareness together with decoding, word reading and non-verbal IQ in a large sample of 703 French first-graders from low-SES families after 3 months of formal instruction (November). Pseudowords and word reading were both assessed because cross-linguistic studies have shown that word reading skills can develop faster in more transparent orthographies than English (Seymour et al., 2003; but see also Moll et al., 2014). We used three oral tasks to assess morphological awareness of derivational morphology. The Relationship Judgment task (e.g., do these words belong to the same morphological family or not? heat-heater;… ham-hammer); the lexical Sentence Completion task [e.g., Someone who runs is a …? (runner)]; and the non-lexical Sentence Completion task [e.g., Someone who lums is a…? (lummer)]. The tasks differ on implicit/explicit demands and also tap different kinds of morphological knowledge. The Judgement task measures the phonological and semantic properties of the morphological relationship and the Sentence Completion tasks measures knowledge of morphological production rules, with the non-lexical version assessing how well these rules can be generalized to novel items. These tasks were very frequently used in studies with first-graders (Carlisle and Nomanbhoy, 1993; Casalis and Louis-Alexandre, 2000; Rispens et al., 2008; Duncan et al., 2009; Wolter et al., 2009; Apel et al., 2013a; Kim et al., 2013; Apel and Diehm, 2014).

### Overview of data analysis

#### Graph modeling analysis

In an exploratory data-driven analysis, data were processed using a graphical modeling approach (Vandenberghe et al., 2013; Massa et al., 2015) which gives crucial information about how skills known to be involved in learning to read are organized in memory. With this method we were able to identify what were the oral language skills involved in the acquisition of word reading skills and how they were related to each other (direct or indirect connections). We found that: (1) listening comprehension skills are at the heart of the reading acquisition process, (2) word reading and decoding skills depend directly on phonemic awareness and indirectly on listening comprehension, (3) the influence of higher order skills (vocabulary, morphological skills, non-verbal capacities) on word reading and decoding is not direct but rather indirect via listening comprehension, (4) morphological and phonological skills, combined to listening comprehension seem to have a directed and acyclic role in the acquisition of word reading and decoding skills (they are ordered into a dependence chain), (5) the components of morphological skills as assessed by our tasks (Relationship Judgment and Sentence Completion) have independent relationships with listening comprehension.

#### Directed acyclic graph analysis

As stated above, the directed relationships may also be put in a parametric form (see Kiiveri and Speed, 1982) and used as the starting point for further analysis with SEM. We thus followed a confirmatory approach to test statistical hypotheses based on substantive theory and/or previous empirical research.

The general model we tested is as follows. Cutting and Scarborough (2012) provided a useful comprehensive general framework of reading comprehension where listening comprehension comprises several language skills such as vocabulary, morphological and syntactic as well as more general skills such as executive function. So, one can expect that the development of listening comprehension skills would be directly influenced by morphological and vocabulary knowledge as well as by non-verbal IQ. The three morphological awareness tasks we used may exert different influence on listening comprehension skills as they tap on different aspects of morphological knowledge.

Duncan et al. (2009) assessed these tasks with first to third French graders. They found that performance of the relationship judgment task was higher than that of the lexical and non-lexical sentence completion tasks. They claimed that it may be because the latter requires a more explicit level of awareness (Gombert, 1992) and also because the relationship judgement task can be done using a semantic strategy to accurately distinguish between word pairs like “heat-heater” and “ham-hammer.” Carlisle and Nomanbhoy (1993) reported findings that support this interpretation. They found that vocabulary accounted for a significant portion of variance of the relationship judgement task performance while that of phoneme awareness was not significant. For the lexical sentence completion task both vocabulary and phoneme awareness contributed significantly, suggesting that this morphological task assessing knowledge of morphological production rules is more heavily constrained by phonological factors. Moreover, Duncan et al. (2009) also reported that the lexical sentence completion task was easier for children than the non-lexical version implying that the altter increased the need for metalinguistic control over morphological knowledge. Fundamentally, the knowledge of morphological production rules measured by the lexical version of the sentence completion should directly influence the level of performance of the non-lexical version.

As pointed in the introduction, there is a huge literature showing that oral language development underpins reading acquisition. Importantly, Nation and Snowling (2004), for example, showed with children aged 8 years that both phonological skills (such as phonological awareness) and children's oral language proficiency as measured by vocabulary, listening comprehension and semantic skills influence the course of word reading development. Interestingly beside phonological skills, semantic abilities may also influence decoding because of their predictive relationship with phonological awareness (Share et al., 1984; Wagner et al., 1993; Burgess and Lonigan, 1998; Lonigan et al., 1998, 2000; Bishop et al., 2004) and because phonological segmentation is stimulated as semantic knowledge increases (Carroll et al., 2003). Therefore, listening comprehension as a proxy of children's oral language proficiency can determine the level of phoneme awareness skills.

However, contrary to the dominant view of word reading acquisition, which states that children's phonological skills are the foundation upon which the decoding ability needed to develop further word reading proficiency is built (Nation and Snowling, 2004), our graphical modeling analysis showed that phoneme awareness influences word reading which in turn influences pseudoword reading (decoding). Following Share (1995, 1999), one can argue that in the very early stages of reading acquisition, when children have few grapheme-phoneme skills, they also can utilize top-down knowledge of word meanings to help with the process of decoding. Thus reading words would boost decoding skills.

This model additionally reports that vocabulary skills influence directly phonological skills. This is in line with Ouellette and Haley (2013)'s study which reported that oral vocabulary in kindergarten predicted unique variance of phonemic awareness into grade 1. These data were interpreted within the Lexical Restructuring Model (LRM, Metsala and Walley, 1998; Walley et al., 2003) which conceives oral vocabulary as the key contributor to phoneme awareness. Vocabulary growth needs phonemic representations of words to be accurate because of increasing number of phonologically similar words. As a consequence, vocabulary acquisition needs restructuring the phonemic level of word representations in the lexicon which in turn develops phoneme awareness.

The model also shows that one component of morphological awareness directly feeds the phoneme awareness skills. This result goes again the dominant view which states that during early reading acquisition, although there may be links between phonological and morphological awareness because both require the manipulation of parts of speech, a portion of morphological development may be dependent on phonological awareness (see Law and Ghesquière, 2017, for recent account). However, Carlisle (1995) and Fowler and Liberman (1995) have suggested that morphological awareness may encompass a larger range of abilities than phonological awareness, some of which may emerge early in development. Critically, the development of phonological awareness takes place in kindergarten and first grade, whereas morphological awareness may develop later in childhood (Mahony, 1994; Leong, 2000). From this point of view, because the development of morphological awareness is delayed relative to phonological awareness, phonological awareness may be extended by morphological awareness. In line with this interpretation, Carlisle (1995) suggests that morphological awareness may foster growth in phonological awareness as children learn to appreciate systematic phonological variations that occur in morphologically related words (i.e., singer, singing, sings).

## Materials and methods

### Participants

At the beginning of the first school year, the sample consisted of 703 French children (371 girls and 332 boys) with a mean age of 6 years 2 months (range: 5 years 9 months to 6 years 9 month). These children were attending 30 different elementary school classes, all in a “Priority Education Area,” which reflects a low socio-economic catchment area exhibiting a variety of social difficulties. Thus, these children belonged to low socio-economic status families, defined according to the Government criteria, as those with high levels of unemployment or whose predominant experience is of low income. In addition, the families frequently are single parent families and often do not have French as the native language. The present study was conducted in accordance with the Declaration of Helsinki. It was conducted with the understanding and the written consent of each child's parent and in accordance with the ethical guidelines between the academic organization (LPNC-CNRS) and educational organizations.

### Assessments

Each child was tested on the following domains: Listening comprehension, word and pseudoword reading skills, vocabulary, morphological awareness skills, phonemic awareness skill, and non-verbal IQ. The children were tested individually. Psychologists, who were trained and regularly supervised on site, were responsible for administering all of the tests. Each testing session for each child lasted ~45 min and took place in a quiet room in the schools.

#### Non-verbal IQ

Non-verbal IQ was assessed with the Progressive Matrices Standard 47 (Raven et al., 1998) in which 36 problems were presented to the children. Each problem consisted in finding the missing part of an incomplete design among the six options provided.

#### Phonemic awareness

The phonemic awareness task was composed of 11 items assessing phonemic segmentation skills (i.e., phonemic awareness). One test with two subtests assessed onset segmentation (6 items, 3 in each subtest): the children had to choose from four options what a reference word beginning with a consonantal cluster would become when (1) the onset is removed (e.g., for “*trois,”* the options were “*deux”/“oie”/“râteau”/“train*,” the correct response being “oie”) and (2) when a part of the onset is removed (e.g., for “*croix,”* the options were “*poire”/“toit”/“noix”/“roi*,” the correct response being “roi”). The other phonemic test involved an oddity task: the children had to spot the odd one out of three words on the basis of the initial phoneme (e.g., “*coq”/“col”/“botte,”* odd word: “*botte,”*) or the final phoneme (e.g., “*car”/”selle”/”pelle,”* odd word: “*car,”*). All words were presented orally together with a picture. The phonology scale had 11 points.

#### Listening comprehension

Listening comprehension was assessed at a syntactic-semantic level (ECoSSe: Lecocq, 1996). In this task, children were shown four pictures and had to choose the one that exactly depicted the situation described in a sentence read by the examiner. The listening comprehension scale had 25 points (one point per item) and the percentage of correct responses was calculated.

#### Vocabulary

Receptive vocabulary was assessed on a standardized vocabulary test (TVAP: Deltour and Hupkens, 1980). The children chose the picture (from six) that illustrated the word read aloud by the experimenter. There were 30 items, each scored on a scale from 0 to 2 (2 points are awarded for the choice of the correct response and 1 point for the approximate response, e.g., the picture of a “big house” for the item “castle”), making for a total possible score of 60 points.

#### Morphological awareness

The morphological awareness skills were assessed using a test in which children had to perform a word relationship judgment task and a word completion task. In the word relationship judgment task 10 word pairs were presented to children who had to determine if the two words belong to the same family or not (e.g., “coureur/courir vs. coureur/courage). In the word completion task 20 sentences were presented to children in which they had to complete either ten words (i.e., lexical completion task) or ten pseudo words (i.e., non-lexical completion task) with a term from the same family (e.g., for the word sentence “*un homme qui coiffe est un…”* the child must answer “*coiffeur”* and for the pseudo word sentence “*un homme qui plude est un…”* the child must answer “*pludeur.”* For all the tasks, a training phase was proposed to the children with 4 trials during which, in case of errors, the children were explained the concept of morphological family (word relationship judgment) or the meaning of the word. suffix (word completion task).

#### Reading

Reading skills were assessed through a 1-min test of words reading. There were 35 words made-up of one to six letters. Decoding skills were assessed through a 1-min test of pseudoword reading. There were 30 pseudowords made-up of one to five letters. The number of words correctly read in 1 min (wpm) was calculated. The words and pseudowords of these reading tasks all have a monomorphemic structure.

### Data analysis plan

In recent years, there has been a strong interest in representing multivariate data as networks. One of these networks, the Markov network, is based on undirected acyclic graphs (Friedman, 2004), whereas directed graphs (directed acyclic graphs, i.e., DAGs) are Bayesian networks. Graph networks come from the process of high-dimensional data and provide a framework for modeling how several variables are mutually related and how conditional independence structures can be represented graphically. Traditional approaches to analyzing data from neuropsychological and behavioral sciences have conducted factor analyses, cluster analyses or multiple regression analyses. Factor analyses are useful for identifying factors that may contribute to several observed variables. Cluster analysis is a statistical technique used to identify groups of entities (observations or variables) that have similar characteristics. Graph analyses extend these approaches by testing the conditional independences of the selected assessments directly, without the need for assumptions about underlying hidden factors. Moreover, graph analysis goes beyond multiple regression analyses by capturing the interactions between all the variables rather than only the linear dependency of one measure on a set of explanatory variables (Massa et al., 2015).

The form of multivariate analysis known as Graphical modeling had its origina in the fields of physics and genetics (Gibbs, 1902; Wright, 1921), and is an approach that combines a statistical model with a mathematical object, a graph. The graph is defined as a pair *G* = (*V, E*), where *V* is a set of *vertices* or *nodes* and *E* is a set of *edges* (Edwards, 2000). Højsgaard et al. (2012) explain that “each edge is associated with a pair of nodes…Edges may in general be directed, undirected or bidirected. Graphs are typically visualized by representing nodes by circles or points, and edges by lines, arrows, or bidirected arrows.” The key tool in graphical modeling is the dependence graph. In the dependence graph the nodes represent random variables of a multivariate distribution. The nodes can be connected by different types of edges which reflect the statistical relations between the variables. For understanding graphical modeling, the notion of conditional independence is crucial. This example by Da et al. (2011) from probability theory helps to explain this concept: “two events *X* and *Y* are conditionally independent given a third event *Z* precisely, if the occurrence or non-occurrence of *X* and the occurrence or non-occurrence of *Y* are independent events in their conditional probability distribution given *Z.”* In other words, if the value of *Z* is known, knowledge of whether *X* or *Y* occurs provides no information of the likelihood of *Y* or *X* occurring, respectively. That said, if two nodes *X* and *Y* are separated in the graph by a nodes *Z*, then the corresponding random variables *X* and *Y* are conditionally independent given *Z*. Importantly, “undirected edges represent associations between random variables and a missing edge reflects the fact that random variables are conditionally independent” (Massa et al. (2015), from their behavioral study using graph modeling). As statistical objects, graphs illustrate the variables, and parameters of models making it possible to read the independence structure of the model directly from the graph.

**Figure 1:**
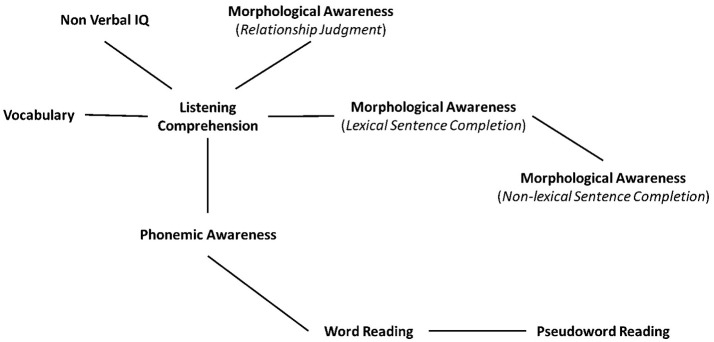
Dependence graph (exploratory and undirected minimal forest) on all variables.

First, in this paper, the focus will be on undirected graphical models, i.e., graphical models where graphs have only undirected edges. In other words, no assumptions will be made about directed relations between any variables. This goal of this approach is to infer the structure of the dependence graph which gives the best description of the conditional independences and associations between all the variables. The undirected graph (i.e., so-called Markov graph) illustrates the correspondence between nodes and the conditional independence relations between all the variables (a Gaussian Markov graph is characterized by zeros in the inverse of the covariance matrix). In this study, to investigate this dependence graph we used the methods and their implementation provided by Højsgaard et al. (2012), by following the pipeline using R with *cran-r* packages. More precisely, we estimated the model parameters of the dependence graph using both the Akaike Information Criterion (AIC) and Bayesian Information Criterion (BIC) forests (Chow and Liu, 1968; Edwards et al., 2010). The former (AIC) is defined as −2 ln *L* + 2*r*, where *L* is the maximized likelihood under the model and *r* is the number of parameters in the model, and the latter (BIC) as −2 ln *L* + ln(*N*)*r*. When working with graphical models, a “forest” or a “tree” (see Edwards et al., 2010, for a detailed distinction between “tree” and “forest” terminologies, but for a general definition, a forest may have several connected components, the trees) corresponds to a graph with *n* nodes and several *k* connected components. In modern terminology, trees and forests are special cases of undirected and acyclic graphs (i.e., graph with no cycles with a topological ordering, i.e., a sequence of the vertices such that every edge is directed from earlier to later in the sequence). To do so, we used the algorithm implemented in the *minForest* function in the *gRapHD-r* package. The graph obtained (see Figure 1 in the Results section) corresponds to a minimal forest undirected and acyclic graph (i.e., exploratory and data-driven graph). This approach can be applied as an initial step toward identifying the overall dependence structure of high-dimensional data with uses including the detection of distinct connected components, neighborhoods, or interesting features, such as hub nodes (Edwards et al., 2010).

**Figure 2:**
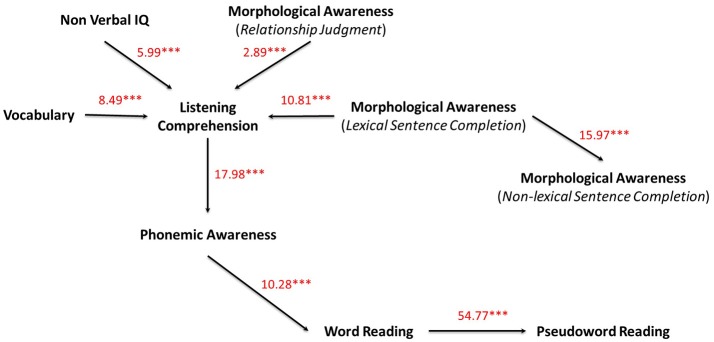
Confirmatory and directed graph (DAG) on all variables. Standardized model parameters (z-transformed regression coefficients) obtained by structural equation method (Latent Variable Analysis Lavaan Package) are depicted on each directed edge with respective *p*-values (^***^p < 0.001).

Second, to address theoretical questions, we applied a DAG and tested measurement and fit models by use of the structural equation modeling (SEM) method on the forest graph (see Figure 2 in the results section). Structural modeling was performed using Latent Variable Analysis with *Lavaan-R* package. Model fitting was based on the correlation matrix (see Table 1) and indicators of fit (χ^2^ statistic) tested whether the model was consistent with the covariation pattern among the observed variables. The χ^2^ statistic measures the model's “badness of fit” compared to a saturated model. However, since the χ^2^ is vulnerable to sample size and/or departure from multivariate normality (χ^2^ tends to be significant when the sample size is large which means that the χ^2^ statistic nearly always rejects the model when large samples are used, see Bentler and Bonnet, 1980), we also evaluated model fit based on different fit indices recommended in the literature (see Finkenauer et al., 1998). We used the Root Mean Square Error of Approximation as a measure for the discrepancy of the fit of the model per *df* used (RMSEA; see Steiger, 1990). The RMSEA also assesses the model's “badness of fit” (i.e., difference between the observed and predicted covariances) and traditionally researchers consider a RMSEA value of ~0.08 or less to indicate a close fit in relation to the *df* (Hu and Bentler, 1998, see also Miyake et al., 2000). The other fit indices (Comparative Fit Index, CFI; Tucker-Lewis index, TLI; and Incremental Fit Index, IFI) are typically used to measure “goodness of fit” with values around or greater 0.90 or 0.95 are indicative of excellent model fit (Kline, 2015). Moreover, the Goodness of fit index (GFI) estimates how well the sample variances and covariances are reproduced by the proposed model. As for the CFI, which measures the covariance in the data reproduced by the model, for GFI, a value greater than 0.90 indicates an acceptable fit. Both AIC and BIC that take the goodness of fit as well as the number of estimated parameters into account were also introduced. After estimation of the parameters of the model, the standardized coefficients were entered on the structural DAG model paths (see Figure 2). The graph obtained corresponds to a minimal forest directed and acyclic graph (i.e., confirmatory and both theory- and graph-driven model). These paths can be considered as regression or path coefficients inferred from the observed correlations. A summary of fit indices for the DAG model is presented in Table 2 in the Results section.

**Table 1:** Empirical partial correlation matrix of the variables (above the diagonal line), and Pearson correlation matrix (below the diagonal line).

	**1**	**2**	**3**	**4**	**5**	**6**	**7**	**8**	**9**
1.Listening Comprehension		0.01	−0.02	0.14^***^	0.27^***^	0.07	0.30^***^	0.12^***^	0.19^***^
2.Word Reading	0.26^***^		0.81^***^	0.11^**^	−0.01	0.01	0.00	0.03	0.09^*^
3.Pseudoword reading	0.26^***^	0.85^***^		0.05	0.00	0.05	0.06	0.03	0.01
4.Phonemic awareness	0.40^***^	0.38^***^	0.36^***^		0.15^***^	0.10^**^	0.03	0.06	0.02
5.Vocabulary	0.55^***^	0.22^***^	0.22^***^	0.38^***^		0.08^*^	0.28^***^	0.04	0.03
6.MA judgment	0.28^***^	0.23^***^	0.24^***^	0.27^***^	0.26^***^		0.01	0.09^*^	0.05
7.MA lexical	0.58^***^	0.28^***^	0.28^***^	0.35^***^	0.54^***^	0.25^***^		0.30^***^	−0.05
8.MA non-lexical	0.43^***^	0.27^***^	0.27^***^	0.31^***^	0.37^***^	0.25^***^	0.51^***^		0.02
9. Non-verbal IQ	0.31^***^	0.26^***^	0.24^***^	0.20^***^	0.20^***^	0.17^***^	0.17^***^	0.18^***^	
mean (*N* = 703)	79.9	6.5	6.2	7.1	37.2	12.6	5.2	2.4	20.5
Standard deviation	15.3	6.5	5.5	2.6	7.4	2.6	2.4	2.1	5.1

* p < 0.05;

** p < 0.01;

*** *p < 0.001*.

*Variables: MA, Morphological awareness; all others are transparent*.

*Means and standard deviations are presented at the bottom of the correlation matrix*.

**Table 2:** Comparison of generalized least square estimations of the three proposed models: The minForest Graph is the exploratory and undirected graphical model; the DAG is the confirmatory (SEM) and directed graph; and the Extended DAG is the theory-driven and directed graph including two direct contributions of Vocabulary and Morphological awareness (lexical sentence completion) to Phonemic awareness.

	***minForest* graph**	**DAG**	**Extended DAG**
χ^2^ (df)	147.01^***^ (19)^T^	174.92^***^ (22)^TT^	127.01^***^ (20)^TT^
AIC	34989.362	34858.334	34824.362
BIC	35100.800	34877.656	34846.444
RMSEA [95% CI]	0.094 [0.088–0.101]	0.094 [0.088–0.110]	0.088 [0.080–0.991]
CFI	0.93	0.91	0.93
GFI	0.94	0.92	0.94
TLI	0.90	0.88	0.90
IFI	0.93	0.91	0.93

*** *p < 0.001*.

T *df in Gaussian Graphical Model = 1/2 {Tr(K'(Σ_0_ – S))} when Y ~ N(0,K^−1^) and K' an estimate of K (the inverse of covariance matrix)*.

TT The number of constraints in the recursive structural model, that is [v*(v+1)/2–p] where v is the number of the observed variables, and p is the number of free parameters.

## Results

Descriptive statistics (means and standard deviations) are presented in Table 1 along with both Pearson correlations among the variables presented below the diagonal line and the empirical partial correlations presented above the diagonal line.

The Empirical partial correlation matrix (above the diagonal line in Table 2) of the variables introduced (i.e., Listening comprehension, Word reading, Pseudoword reading, Phonemic awareness, Vocabulary, Morphological awareness (MA relationship judgment), Morphological awareness (MA lexical sentence completion), Morphological awareness (MA non-lexical sentence completion), and non-verbal IQ, reflects the pairwise correlation of the variables after taking into account all the remaining ones in the domain. It is note-worthy that while all the Pearson correlations are highly significant (all *p*s < 0.001), only a few partial correlations are significant. Among them, a strong coefficient (around 0.30; *p* < 0.001) was found between Listening comprehension and Vocabulary tasks (*r* = 0.27; *p* < 0.001), Listening comprehension and MA lexical sentence completion (*r* = 0.30; *p* < 0.001), Word reading and Pseudoword reading (*r* = 0.81; *p* < 0.001), MA lexical and non-lexical sentence completion (*r* = 0.30; *p* < 0.001), and Vocabulary and MA lexical sentence completion (*r* = 0.28; *p* < 0.001).

### MinForest (undirected graph) measurement model

Figure 1 displays the estimated dependence minimal forest undirected and acyclic graph. Diagrammatic and idiosyncratic graph representations were removed for clarity and we presented nodes (or vertices) without circles or points but only with their respective task's name. However, edges are still represented by lines.

Here we illustrate how to interpret the graph with an example. The graph in Figure 1 shows that node (or vertex) “Vocabulary” and “MA lexical sentence completion” are separated from node “Phonemic awareness” by node “Listening comprehension.” This indicates that “Vocabulary” and “MA lexical sentence completion” are conditionally independent of “Phonemic awareness,” given “Listening comprehension.” Overall, the estimated dependence graph shows that most variables are associated with Listening comprehension. More precisely, Vocabulary, non-verbal IQ, MA relationship judgment, MA lexical sentence completion, and Phonemic awareness tasks were directly connected to Listening comprehension task. These associations imply that the overall Listening comprehension score is related to these 5 factors. Interestingly, when the Listening comprehension score was controlled for, there were no direct connections between MA relationship judgment and MA lexical sentence completion, suggesting some distinction between the processes involved in each tasks, as well as between Vocabulary and Phonemic awareness, suggesting a distinction between oral semantic skills (as estimated by the vocabulary task) and more abstract-level phonological processing skills (as estimated by the phonemic awareness task). In the same line, there was also no relation between Listening comprehension and Word reading when Phonemic awareness is controlled for. The *minForest* graph displayed in Figure 1 is a model of 26 parameters, 9 nodes and 8 edges, with a likelihood ratio (LH; −2^*^Log-likelihood) of 34937.36 (AIC = 34989.362; BIC = 35100.800). The data-driven *minForest* undirected graph demonstrated acceptable model fit (see Table 2), with χ^2^_(19)_ = 147.01; *p* < 0.001; CFI = 0.93; GFI = 0.94; TLI = 0.90; IFI = 0.93; and RMSEA [CI 95%] = 0.094 [0.088–0.101], although for excellent model fit, values greater than 0.90, even 0.95 for both TLI and IFI, and value below 0.08 for RMSEA, are recommended.

### DAG measurement model

Based on the *minForest* graph and in the aim to address theoretical questions, we applied a DAG and tested measurement and fit model by use of recursive structural equation modeling (SEM) method (see Kiiveri and Speed, 1982). The confirmatory and directed graph (DAG) is displayed in Figure 2.

Results of the confirmatory and directed graph demonstrated acceptable model fit, with χ^2^_(22)_ = 174.92; *p* < 0.001; CFI = 0.91; GFI = 0.92; TLI = 0.88; IFI = 0.91; and RMSEA [95% CI] = 0.094 [0.088–0.110]. A summary of fit indices for the DAG model, along with the *minForest* model, is presented in Table 2. AIC and BIC relative to the DAG model were 34858.334 and 34877.656, respectively. Standardized coefficients were placed directly on the paths of the structural diagram. These path coefficients are inferred from the observed correlations, and the significance level of each parameter is indicated (all *p*s < 0.001). The standardized coefficients were ranged from 2.89 (MA relationship 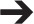 judgment Listening comprehension connection) to 54.77 (Word reading 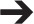 Pseudoword reading connection). Results also showed a strong connection between MA lexical sentence completion 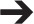 Listening comprehension (10.81) but also 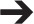 MA non-lexical sentence completion (15.97), and likewise between Listening comprehension 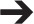 Phonemic awareness (17.98) and between Phonemic awareness 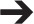 Word reading (10.28). Interestingly, although highly significant, the connection between Vocabulary 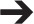 Listening comprehension (8.49) was highest than those between MA relationship judgment 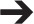 Listening comprehension (10.81), suggesting that morphological awareness skills are highly predictive of listening comprehension skills in First grade readers.

### Extended DAG measurement model

**Figure 3:**
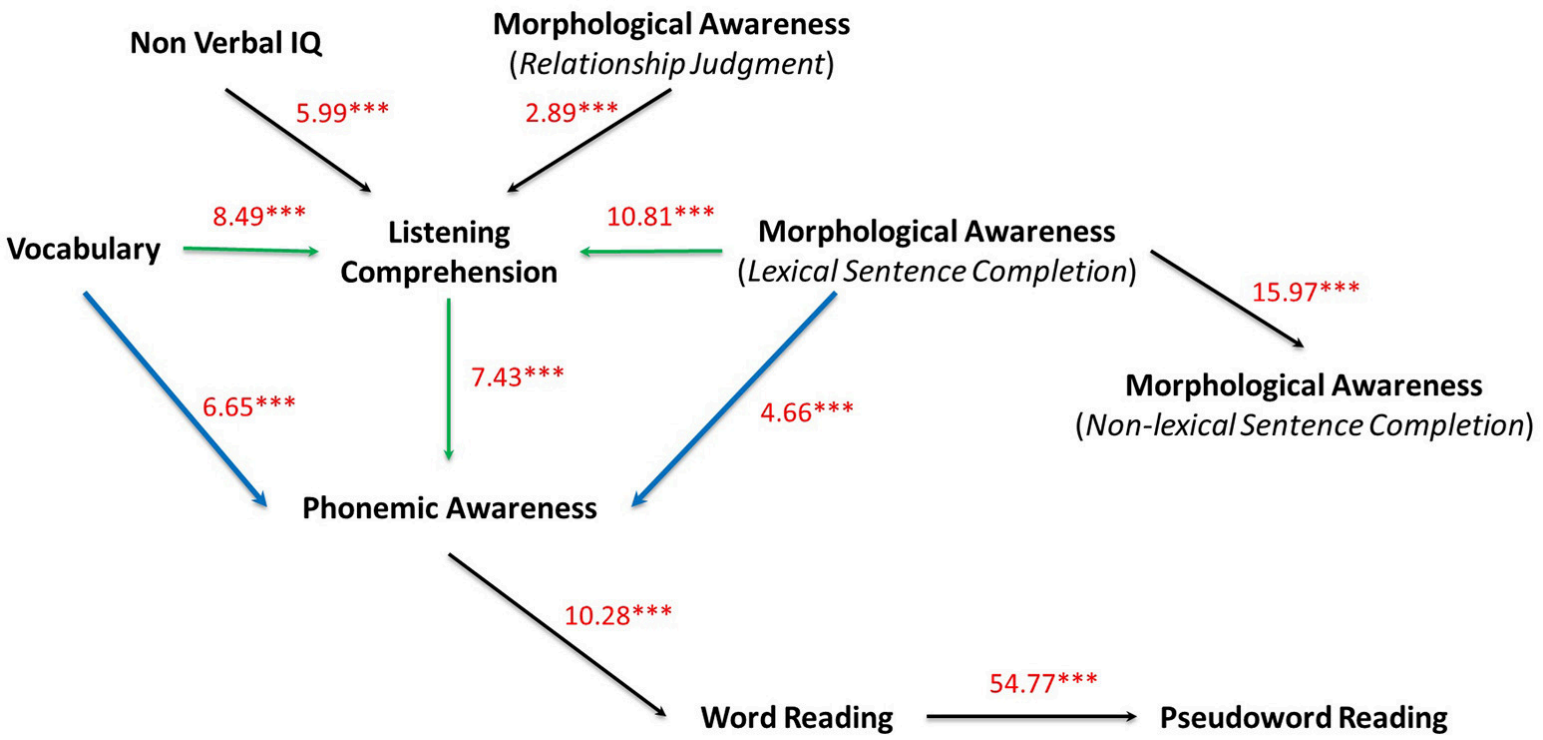
Confirmatory and directed Extended graph (DAG) on all variables. Direct paths between Vocabulary and MA lexical sentence completion to Phonemic awareness are depicted in blue, indirect paths are depicted in green. Standardized model parameters (z-transformed regression coefficients) obtained by structural equation method are depicted on each directed edge with respective *p*-values (^***^p < 0.001).

As can be seen in Table 2, the χ^2^ statistic for both the *minForest* and DAG models did not reach *p* > 0.05 (especially because we had a large sample size) but some fit indices indicated an acceptable fit. To improve the global fit, we re-specified the initial model on the base of the modification indices (MI). A modification index is of use in seeking out a better model. Statistically, this index is a 1-df Chi-square test that indicates the extent to which the discrepancy between model and data can be reduced, as defined by a general fit function, when one parameter is added or freed or when one equality constraint is relaxed (Sorbom, 1986). These MIs suggest links to change in our structure. In other words, the large value indicates that more information can be gained from the data by introducing additional parameters into the model. According to the common SEM practice, these new parameters have to be justified by theoretical models (see the Introduction section). Therefore, in this section, we pursue a confirmatory and theoretically driven model-testing strategy which involves the addition of paths and the evaluation of changes in fit across tested models, consistent with common SEM practice (Kline, 2015). Following the confirmatory DAG model, theoretically plausible alternative were tested with the goal of identifying the most parsimonious and well-fitting model. Figure 3 displays the “Extended DAG model.” In this model, two direct paths have been added, one between nodes Vocabulary and Phonemic awareness, and one between nodes MA lexical sentence completion and Phonemic awareness. These two direct paths are directed to explain performances on Phonemic awareness task (depicted as blue arrows in Figure 3). These two direct paths resulted in different connections strength with a standardized coefficient of: 6.65 between Vocabulary 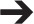 Phonemic awareness; 7.43 between Listening comprehension 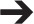 Phonemic awareness; and 4.66 between MA lexical sentence completion 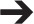 Phonemic awareness.

To summarize the results obtained, a summary of fit indices for Extended DAG model, the DAG model, along with the *minForest* model, is presented in Table 2. As compared to the minForest and DAG models, results were more favorable for the Extended DAG model. First, the discrepancy per *df* is close to the recommended value of 0.08 (Miyake et al., 2000) with RMSEA of 0.088 and 95% CI [0.080–0.991]. Second, the χ^2^ of the Extended DAG model is lower than for the *minForest* and DAG models representing a better fit [Δχ^2^_(2)_ = 174.92–127.01; *p* < 0.001]. Third, all the fit indices indicated an acceptable fit: CFI = 0.93; GFI = 0.94; TLI = 0.90; IFI = 0.93. and finally, AIC and BIC indices for the Extended DAG model (34824.362, and 34846.444, respectively), suggest that the Extended DAG model is the most parsimonious and well-fitting model, since a model that yields the smallest value of AIC is considered best (see for example Finkenauer et al., 1998).

## Discussion

The goal of this study was to investigate the involvement of morphological knowledge in the very early stages of reading acquisition, before reading comprehension can be assessed, among a very large sample of 703 French first-graders from low SES families. In particular, we wanted to identify the network of oral language skills (vocabulary, listening comprehension, phoneme awareness, morphological awareness) that influenced the acquisition of decoding (pseudoword naming) and word recognition (word naming) abilities. In doing so, we have introduced the use of a graphical modeling approach to the data. This powerful method allows examination of how these skills are directly and indirectly related; the potential correlational relationships between all these skills being taken into account at the same time. As advocated by Kiiveri and Speed (1982), we followed up the graphical modeling approach using SEM analysis, which allowed us to refine further our theoretical framework of the very early stages of reading. To our knowledge, this particular statistical analysis has never been applied to data collected on reading acquisition skills, and this may explain some of the new conclusions it appears to bring to the field.

### Which oral language skills Are involved in the early stages of low SES reading acquisition?

The results show that a set of oral language skills are involved, directly or indirectly, in the very early stages of decoding and word reading acquisition. In the results we obtained, listening comprehension is at the heart of this acquisition (this variable is called an unshielded collider in graphical modeling terminology, see Edwards, 2000). This has been often claimed (Hulme and Snowling, 2013) but has only been tested indirectly using regression analyses which control for verbal ability (for example, Lonigan et al., 2000). Some studies have also shown that one component of listening comprehension skills, namely vocabulary, predicted significant variance in irregular word reading in first grade, even after phonemic awareness and decoding were controlled for Ouellette and Beers (2010). Our graphical modeling approach showed that when all the correlations between all of the oral language components used in our study are taken into account, listening comprehension exerts a direct influence on the development of the main predictor of decoding and word reading skills, which is phoneme awareness (Law and Ghesquière, 2017).

We also found that phoneme awareness directly influenced visual word recognition, which in turn directly influenced decoding skills. This set of results may at first glance appear contradictory with a large body of evidence reporting that word decoding skills enhanced by phoneme awareness is one foundation of word reading skills (see for a review Ouellette and Beers, 2010; Sprenger-Charolles and Colé, 2013). However, it is important to remember here that the children in this sample are in the first 3 months of formal reading instruction and their grapheme-phoneme conversion skills may be too limited to read words on the basis of phonological information alone. As a result, lexical knowledge is likely to be involved in the early stages of the acquisition of the decoding process, as suggested in the graphical modeling analysis which showed that word reading skills exerted a direct and sole influence on pseudoword reading skills. Furthermore, at this very early stage of acquisition, all of the processes in the acquisition of decoding skills would appear to be dependent on lexical knowledge as the analysis also demonstrated that both listening comprehension and vocabulary have a direct influence on the development of phoneme awareness (and also morphological awareness). Ouellette and Beers (2010) reported that vocabulary and phoneme awareness explained unique variance of word reading skills in first grade but they did not show how these two skills exert their influence. Note that vocabulary also has an indirect influence on phoneme awareness via listening comprehension skills. Thus as Ouellette and Beers (2010), Metsala and Walley (1998) and Walley et al. (2003) (but see also, Hulme et al., 2002; Nation, 2008) claimed, vocabulary is a powerful determinant of the development of phoneme awareness, because vocabulary growth needs more accurate phonemic representations of words in order to avoid misleading recognition of very phonologically similar words. This process (accurate phonemic analysis) directly impacts the development of phoneme awareness.

Our data indicate that oral language skills are central to the very earliest phases of reading acquisition and expand upon the SVR model that considers word recognition and oral language comprehension skills to be distinct abilities. Our results show that they are not: decoding and word reading were influenced by the oral language skills examined in our study via phoneme awareness. However, contrary to Ouellette and Beers (2010) vocabulary did not exert a direct influence on word reading but rather operated indirectly via phoneme awareness. As explained above, this may be due to the fact that the children in the present study were at the very beginning of using decoding procedures. Thus we would like to outline the dominant position (for example, Hulme and Snowling, 2013), according to which semantic/lexical knowledge may only influence reading comprehension but rather think it will also influence word reading acquisition strongly although indirectly. This is in line with Laing and Hulme (1999) suggesting that lexical knowledge would be involved in very early reading acquisition, when they demonstrated that young children's efficiency at learning written abbreviations for words in the earliest stages of reading was dependent upon the imageability of those words.

### What influence does morphological awareness have in the early stages of low SES reading acquisition?

Our data show that the components of morphological awareness tested (Relationship Judgment and Sentence Completion) are related independently to listening comprehension and are not directly connected to each other. The Relationship Judgment task was performed better than the Sentence Completion task (both lexical and non-lexical versions). This confirms the findings of Duncan et al. (2009) with both English-speaking and French-speaking first graders and suggests that these tasks measure different aspects of morphological knowledge. Indeed, Carlisle and Nomanbhoy (1993) demonstrated that these two tasks were explained by different factors: whereas Relationship Judgment was only explained by vocabulary, Sentence Completion was explained by both vocabulary and phoneme awareness. This is consistent with the observed influence of Sentence Completion on phoneme awareness in the present study. In line with Carlisle (1995), this direct relationship suggests that morphological awareness may foster growth in phonemic awareness as children learn to detect systematic phonological variations occurring in morphologically-related words, which are semantically motivated (as is the case in words sharing derivational affixes: suffixes and prefixes). Casalis and Colé (2009) reported results that partly support this interpretation from a training study with kindergarteners using phonological training, morphological training and also a control group, who did not receive any training. Oral language awareness tasks were used for the training and all three groups received pre- and post-tests in phonological and morphological awareness in order to study the potential transfer effects of the training (from morphology to phonology and vice versa). While phonological training helped children to segment speech into morphemic constituents (morphemic segmentation task), it did not have any effect on the derivational process itself (lexical production task). Likewise, morphological awareness training was not found to help children to segment words into phonemes but it did improve their sensitivity to sound (phoneme oddity task).

Of course, one cannot exclude the possibility that the explicit/implicit dimension differences can also explain some of the differences in task performance (Duncan et al., 2009). Moreover, as we hypothesized, the non-lexical sentence completion task is dependent on the morphological knowledge needed to perform the lexical version of the task. The more explicit manipulation of morphological knowledge required in the non-lexical version made the task more difficult as the results showed lower performance levels in the non-lexical task (for similar results, see Duncan et al., 2009).

Our results also show that morphological awareness has only an indirect influence on word reading skills through phoneme awareness and listening comprehension. The lack of a direct contribution from morphological awareness to word reading is consistent with results from typically developing French first-graders (Casalis and Louis-Alexandre, 2000) and from English-speaking first-graders from low SES families (Apel et al., 2013b). One can hypothesize that this result arose because children were in the very early stages of reading acquisition. Although one cannot exclude the possibility that the particular morphological tasks used may be partly responsible for this result (Apel et al., 2013b), it is important to note that we chose two morphological awareness tasks that had previously been shown by Carlisle and Nomanbhoy (1993) to explain variance in first grade word reading. So the interpretation we consider is that when a large range of oral language tasks is used (as it is our case), the contribution of each of skills might be spread over all the skills taken into account so that it would impact their own contribution.

Finally, we found that vocabulary and morphological awareness made independent contributions to word reading. For both variables, these contributions came via indirect links to listening comprehension as well as via direct links to phonemic awareness. This contrasts with numerous studies that have found that morphological awareness and vocabulary are strongly linked (e.g., Nagy et al., 2006; Sparks and Deacon, 2015). Ramirez et al. (2013) reported that kindergarten morphological awareness was related reciprocally to vocabulary; each made an independent contribution to development in the other. One reason for this discrepancy might be due to the low levels of vocabulary of the low SES children in our study (see Bara et al., 2007 for a more detailed report), which may have prevented direct links with morphological awareness. This would be in line with Spencer et al. (2015) who claimed that “*morphological awareness is an integral part of vocabulary knowledge and may even be considered an additional facet of an individual's depth of knowledge*” (p. 980). But another possibility is that, according to Tighe and Schatschneider (2015) using CFA to explore the relationship between vocabulary knowledge and morphological awareness in adult students, because the construct of morphological awareness is multidimensional, some aspects of this construct would be separate factors from vocabulary knowledge. So more researches are needed to further investigate these relationships.

Future research will also need to answer to one of the limitations of our study that did not take into account the potential influence of plurilingualism of the families of the children. Because, as Ramirez et al. (2013), pointed out in socially disadvantaged areas, linguistic and cultural minorities are often highly represented.

To conclude, our results show that the influence of higher-order skills (vocabulary, morphological awareness, non-verbal capacities) on the earliest phases of word reading and decoding is indirect via listening comprehension. This finding allows two apparently contradictory claims to be reconciled, namely, semantic factors influencing only reading acquisition vs. influencing word reading skills. Additionally, our results also raise concerns about the need for careful reporting of the timing of assessments during the first year of formal reading instruction when word reading and decoding develop so quickly.

The graphical modeling approach has enabled us to produce a clear picture of the network of oral language skills involved at the very outset of low SES reading acquisition. Our data showed that vocabulary knowledge and morphological awareness can be very powerful factors in the emergence of phoneme awareness. While most training studies have focused on only one of these skills (Apel et al., 2013a; Kim et al., 2013; Apel and Diehm, 2014), Ramirez et al. (2013) trained both skills in kindergarteners from low SES backgrounds but, nevertheless, were unable to identify their impact on the development of word reading skills. Morphological awareness tasks offer the means to tap the development of knowledge about both oral and written language. The Relationship Judgement task assesses awareness of the semantic relationship between words and as such is a powerful tool in learning new words from spoken language. The lexical Sentence Production task was also found to influence listening comprehension but interestingly also enhanced phoneme awareness. Therefore, our study indicates that morphology can have varied and specific influences on reading acquisition among low SES groups.

The results of this study as well as those of Apel et al. (2013) show the importance of training morphological skills in children from low SES backgrounds. In France, in the reading manual Crocolivre (Gombert et al., 2000-2002) for children from 1st grade to 2nd grade, systematically teaches the morphology of words. This teaching should therefore be systematized from the beginning of the first grade.

## Author contributions

PC: Experiment design, data collection and analysis, writing article; EC: Data analysis, writing article; LD: Writing article; AT: Data analysis; EG and LS-C: Experiment design, data collection; AE-A: Data analysis, writing article.

### Conflict of interest statement

The authors declare that the research was conducted in the absence of any commercial or financial relationships that could be construed as a potential conflict of interest.

## References

[B1] Anglin J. M. (1993). Vocabulary development: a morphological analysis. Monogr. Soc. Res. Child Dev. 58, 1–186. 10.2307/1166112

[B2] Apel K. Lawrence J. (2011). Contributions of morphological awareness skills to word-level reading and spelling in first-grade children with and without speech sound disorder. J. Speech Lang. Hear. Res. 54, 1312–1327. 10.1044/1092-4388(2011/10-0115) 21386040

[B3] Apel K. Brimo D. Diehm E. Apel L. (2013a). Morphological awareness intervention with kindergartners and first- and second-grade students from low economic status homes: a feasibility study. Lang. Speech Hear. Serv. Sch. 44, 161–173. 10.1044/0161-1461(2012/12-0042) 23633641

[B4] Apel K. Diehm E. (2014). Morphological awareness intervention for kindergartners and first and second grade students from low SES homes: a small efficacy study. J. Learn. Disabil. 47, 65–75. 10.1177/0022219413509964 24191977

[B5] Apel K. Diehm E. Apel L. (2013b). Using multiple measures of morphological awareness to assess its relation to reading. Top. Lang. Disord. 33, 42–56. 10.1097/TLD.0b013e318280f57b

[B6] Bara F. Gentaz E. Colé P. (2007). Haptics in learning to read with children coming from low socio-economic status families. Br. J. Dev. Psychol. 25, 643–663. 10.1348/026151007X186643

[B7] Bentler P. M. Bonnet D. C. (1980). Significance tests and goodness of fit in the analysis of covariance structures. Psychol. Bull. 88, 588–606.

[B8] Bishop D. V. M. Adams C. V. Norbury C. F. (2004). Using nonword repetition to distinguish genetic and environmental influences on early literacy development: a study of 6-year-old twins. Am. J. Med. Genet. B Neuropsychiatr. Genet. 129, 94–96. 10.1002/ajmg.b.30065 15274048

[B9] Bollen K. A. (1989). Structural Equations with Latent Variables. Ville: Wiley-Blackwell.

[B10] Bowers P. N. Kirby J. R. Deacon S. H. (2010). The effects of morphological instruction on literacy skills: a systematic review of the literature. Rev. Educ. Res. 80 144–179. 10.3102/0034654309359353

[B11] Burgess S. R. Lonigan C. J. (1998). Bidirectional relations of phonological sensitivity and pre-reading abilities: evidence from a preschool sample. J. Exp. Child Psychol. 70, 117–141. 10.1006/jecp.1998.2450 9729452

[B12] Cabell S. Q. Justice L. M. Logan J. A. R. Konold T. R. (2013). Emergent literacy profiles among prekindergarten children from low-SES backgrounds: longitudinal considerations. Early Child. Res. Q. 28, 608–620. 10.1016/j.ecresq.2013.03.007

[B13] Carlisle J. F. (1995). Morphological awareness and early reading achievement, in Morphological Aspects of Language Processing, ed Feldman L. B. (Hillsdale, NJ: Erlbaum), 189–209.

[B14] Carlisle J. F. (2003). Morphology matters in learning to read: a commentary. Read. Psychol. 24, 291–322. 10.1080/02702710390227369

[B15] Carlisle J. F. Nomanbhoy D. M. (1993). Phonological and morphological awareness in first graders. Appl. Psycholinguist. 14, 177–195. 10.1017/S0142716400009541

[B16] Carlisle J. F. Stone C. A. (2005). Exploring the role of morphemes in word reading. Read. Res. Q. 40, 428–449. 10.1598/RRQ.40.4.3

[B17] Carroll J. M. Snowling M. J. Hulme C. Stevenson J. (2003). The development of phonological awareness in preschool children. Dev. Psychol. 39, 913–923. 10.1037/0012-1649.39.5.913 12952403

[B18] Casalis S. Colé P. (2009). On the relationship between morphological and phonological awareness: effects of training in kindergarten and in first-grade reading. First Lang. 29, 113–142. 10.1177/0142723708097484

[B19] Casalis S. Louis-Alexandre M.-F. (2000). Morphological analysis, phonological analysis and learning to read French: a longitudinal study. Read. Writ. 12, 303–335. 10.1023/A:1008177205648

[B20] Chow C. Liu C. (1968). Approximating discrete probability distributions with dependence trees. Inf. Theory IEEE Trans. 14, 462–467. 10.1109/TIT.1968.1054142

[B21] Cutting L. E. Scarborough H. S. (2012). Multiple Bases for comprehension difficulties: the potential of cognitive and neurobiological profiling for validation of sub- types and development of assessments, in Reaching an Understanding, eds Sabatini J. O'Reilly T. Albro E. R. (Lanham, MD: Rowman & Littlefield Education), 101–116.

[B22] Da M. Wei W. Hai-guang H. Jian-he G. (2011). The application of Bayesian classification theories in distance education system. I. J. Mod. Educ. Comput. Sci. 4, 9–16. 10.5815/ijmecs.2011.04.02

[B23] Deacon H. S. (2012). Sounds, letters and meanings: the independent influences of phonological, morphological and orthographic skills on early word reading accuracy. J. Res. Read. 35, 456–475. 10.1111/j.1467-9817.2010.01496.x

[B24] Deacon S. H. Kirby J. R. (2004). Morphological awareness: just “more phonological”? The roles of morphological and phonological awareness in reading development. Appl. Psycholinguist. 25, 223–238. 10.1017/S0142716404001110

[B25] Deltour J. J. Hupkens D. (1980). Test de Vocabulaire Actif et Passif Pour Les Enfants (5 à 8 ans). Issy-les-Moulineaux: E.A.P.

[B26] Dempster A. P. (1972). Covariance selection. Biometrics 28, 157–175. 10.2307/2528966

[B27] Dollaghan C. A. Campbell T. F. Paradise J. L. Feldman H. M. Janosky J. E. Pitcairn D. N.. (1999). Maternal education and measures of early speech and language. J. Speech Lang. Hear. Res. 42, 1432–1443. 10.1044/jslhr.4206.1432 10599625

[B28] Duncan L. G. Seymour P. H. (2000). Socio-economic differences in foundation-level literacy. Br. J. Psychol. 91, 145–166. 10.1348/000712600161736 10832511

[B29] Duncan L. G. Casalis S. Colé P. (2009). Early metalinguistic awareness of derivational morphology: observations from a comparison of English and French. Appl. Psycholinguist. 30, 405–440. 10.1017/S0142716409090213

[B30] Edwards D. (2000). Introduction to Graphical Modelling. New York, NY: Springer.

[B31] Edwards D. De Abreu G. C. Labouriau R. (2010). Selecting high-dimensional mixed graphical models using minimal AIC or BIC forests. BMC Bioinformatics 11:18. 10.1186/1471-2105-11-18 20064242 PMC2823705

[B32] Fernald A. Marchman V. A. Weisleder A. (2013). SES differences in language processing skill and vocabulary are evident at 18 months. Dev. Sci. 16, 234–248. 10.1111/desc.12019 23432833 PMC3582035

[B33] Finkenauer C. Luminet O. Gisle L. El Ahmadi A. van der Linden M. Philippot P. (1998). Flashbulb memories and the underlying mechanisms of their formation: towards an Emotional-Integrative Model. Mem. Cogn. 26, 516–531. 10.3758/BF03201160 9610122

[B34] Fluss J. Ziegler J. C. Warszawski J. Ducot B. Richard G. Billard C. (2009). Poor reading in French elementary school: the interplay of cognitive, behavioral, and socioeconomic factors. J. Dev. Behav. Pediatr. 30, 206–216. 10.1097/DBP.0b013e3181a7ed6c 19412126

[B35] Fowler A. E. Liberman I. Y. (1995). The role of phonology and orthography in morphological awareness, in Morphological aspects of language processing, ed Feld-man L. B. (Hillsdale, NJ: Erlbaum), 157–188.

[B36] Friedman N. (2004). Inferring cellular network using probabilistic graphical models. Science 33, 799–805. 10.1126/science.1094068 14764868

[B37] Gentaz E. Sprenger-Charolles L. Theurel A. (2015). Differences in the predictors of reading comprehension in first graders from low socio-economic status families with either good or poor decoding skills. PLoS ONE 10:e0119581 10.1371/journal.pone.0119581 25793519 PMC4368721

[B38] Gentaz E. Sprenger-Charolles L. Theurel A. Colé P. (2013). Reading comprehension in a large cohort of French first graders from low socio- economic status families: a 7-month longitudinal study. PLoS ONE 8:e78608. 10.1371/journal.pone.0078608 24250802 PMC3826761

[B39] Gibbs J. W. (1902). Elementary Principles in Statistical Mechanics. Woodbridge, CT: Ox Bow Press.

[B40] Gombert J. E. (1992). Metalinguistic Development. London: Harvester Wheatsheaf.

[B41] Gombert J. E. Colé P. Desvignes J. Gaberel A. Sonnet J. Valdois S. (2000-2002). Crocolivre CP& CP-CE1. Paris: Nathan.

[B42] Gough P. Tunmer W. (1986). Decoding, reading, and reading disability. Remedial Spec. Educ. 7, 6–10. 10.1177/074193258600700104

[B43] Hemmerechts K. Agirdag O. Kavadias D. (2017). The relationship between parental literacy involvement, socio-economic status and reading literacy. Educ. Rev. 69, 85–101. 10.1080/00131911.2016.1164667

[B44] Hoff E. (2006). How social contexts support and shape language development. Dev. Rev. 26, 55–88. 10.1016/j.dr.2005.11.002

[B45] Hoff E. (2012). Interpreting the early language trajectories of children from low-SES and language minority homes: implications for closing achievement gaps. Dev. Psychol. 49, 4–14. 10.1037/a0027238 22329382 PMC4061698

[B46] Højsgaard S. Edwards D. Lauritzen S. L. (2012). Graphical Models With R. New York, NY: Springer.

[B47] Hoover W. A. Gough P. B. (1990). The simple view of reading. Read. Writ. Interdisc. J. 2, 127–160. 10.1007/BF00401799

[B48] Hu L. Bentler P. M. (1998). Fit indices in covariance structure modeling: sensitivity to underparametrized model misspecification. Psychol. Methods 3, 424–453. 10.1037/1082-989X.3.4.424

[B49] Hulme C. Snowling M. J. (2013). Learning to read: what we know and what we need to understand better. Child Dev. Perspect. 7, 1–5. 10.1111/cdep.12005 26290678 PMC4538787

[B50] Hulme C. Hatcher P. J. Nation K. Brown A. Adam J. Stuart G. (2002). Phoneme awareness is a better predictor of early reading skills than onset-rime awareness. J. Exp. Child Psychol. 82, 2–28. 10.1006/jecp.2002.2670 12081455

[B51] Huttenlocher J. Vasilyeva M. Cymerman E. Levine S. (2002). Language input and child syntax. Cogn. Psychol. 45, 337–374. 10.1016/S0010-0285(02)00500-5 12480478

[B52] Huttenlocher J. Waterfall H. Vasilyeva M. Vevea J. Hedges L. V. (2010). Sources of variability in children's language growth. Cogn. Psychol. 61, 343–365. 10.1016/j.cogpsych.2010.08.002 20832781 PMC2981670

[B53] Keenan J. M. Betjemann R. S. Olson R. K. (2008). Reading comprehension tests vary in the skills they assess: differential dependence on decoding and oral comprehension. Sci. Stud. Read. 12, 281–300. 10.1080/10888430802132279

[B54] Kiiveri H. Speed T. P. (1982). Structural analysis of multivariate data: a review. Sociol. Methodol. 13, 209–289. 10.2307/270722

[B55] Kieffer M. J. (2013). Morphological awareness and reading difficulties in adolescent Spanish-speaking language minority learners and their classmates. J. Learn. Disabil. 47, 44–53. 10.1177/0022219413509968 24219915

[B56] Kim Y. S. Apel K. Al Otaiba S. (2013). The relation of linguistic awareness and vocabulary to word reading and spelling for first- grade students participating in response to intervention. Lang. Speech Hear. Serv. Sch. 44, 337–348. 10.1044/0161-1461(2013/12-0013) 23833281 PMC3852899

[B57] Kirby J. R. Savage R. S. (2008). Can the simple view deal with the complexities of reading? Literacy 42, 75–82. 10.1111/j.1741-4369.2008.00487.x

[B58] Kirby J. R. Deacon S. H. Bowers P. N. Izenberg L. Wade-Woolley L. Parrila R. (2012). Children's morphological awareness and reading ability. Read. Writ. 25, 389–410. 10.1007/s11145-010-9276-5

[B59] Kirby J. R. Desrochers A. Roth L. Lai S. V. (2008). Longitudinal predictors of word reading development. Can. Psychol. 49, 103–110. 10.1037/0708-5591.49.2.103

[B60] Kline R. B. (2015). Principles and Practice of Structural Equation Modeling. New York, NY: The Guilford Press.

[B61] Kruk R. S. Bergman K. (2013). The reciprocal relations between morphological processes and reading. J. Exp. Child Psychol. 114, 10–34. 10.1016/j.jecp.2012.09.014 23123144

[B62] Laing E. Hulme C. (1999). Phonological and semantic processes influence beginning readers' ability to learn to read words. J. Exp. Child Psychol. 73, 183–207. 10357872 10.1006/jecp.1999.2500

[B63] Landerl K. Ramus F. Moll K. Lyytinen H. Leppanen P. H. Lohvansuu K.. (2013). Predictors of developmental dyslexia in European orthographies with varying complexity. J. Child Psychol. Psychiatry 54, 686–694. 10.1111/jcpp.12029 23227813

[B64] Lauritzen S. L. (1996). Graphical Models. Oxford: Oxford University Press.

[B65] Law J. M. Ghesquière P. (2017). Early development and predictors of morphological awareness: disentangling the impact of decoding skills and phonological awareness. Res. Dev. Disabil. 67, 47–59. 10.1016/j.ridd.2017.05.003 28641146

[B66] Lecocq P. (1996). L'E.CO.S.SE: Une épreuve de Compréhension Syntaxico-Sémantique [A Syntactical Semantic Comprehension Test]. Villeneuve d'Ascq: Presses Universitaires du Septentrion.

[B67] Leong C. K. (2000). Rapid processing of base and derived forms of words and grades 4, 5, and 6 children's spelling. Read. Writ. 12, 277–302. 10.1023/A:1008168902922

[B68] Levesque K. C. Kieffer M. J. Deacon S. H. (2017). Morphological awareness and reading comprehension: examining mediating factors. J. Exp. Child Psychol. 160, 1–20. 10.1016/j.jecp.2017.02.015 28364575

[B69] Lonigan C. J. Burgess S. R. Anthony J. L. (2000). Development of emergent literacy and early reading skills in preschool children: evidence from a latent variable longitudinal study. Dev. Psychol. 36, 596–613. 10.1037/0012-1649.36.5.596 10976600

[B70] Lonigan C. J. Burgess S. R. Anthony J. L. Barker T. (1998). Development of phonological sensitivity in 2- to 5-year old children. J. Educ. Psychol. 90, 294–311. 10.1037/0022-0663.90.2.294

[B71] Mahony D. L. (1994). Using sensitivity to word structure to explain variance in high school and college level reading ability. Read. Writ. 6, 19–44. 10.1007/BF01027276

[B72] Malave V. (2008). Graphical Models for Functional Connectivity. Available online at: http://www.cogsci.ucsd.edu/media/uploads/grad/malave-first-draft-prospectus.pdf

[B73] Massa M. S. Wang N. Bickerton W. L. Demeyere N. Riddoch M. J. Humphreys G. W. (2015). On the importance of cognitive profiling: a graphical modelling analysis of domain-specific and domain-general deficits after stroke. Cortex 71, 190–204. 10.1016/j.cortex.2015.06.006 26232552

[B74] Metsala J. L. Walley A. C. (1998). Spoken vocabulary growth and the segmental restructuring of lexical representations: precursors to phonemic awareness and early reading ability, in Word Recognition in Beginning Literacy, eds Metsala J. L. Ehri L. C. (Mahwah, NJ: Erlbaum), 89–120.

[B75] Miyake A. Friedman N. P. Emerson M. J. Witzki A. H. Howerter A. Wager T. D. (2000). The unity and diversity of executive functions and their contributions to complex “frontal lobe” tasks: a latent variable analysis. Cogn. Psychol. 41, 49–100. 10.1006/cogp.1999.0734 10945922

[B76] Moll K. Ramus F. Bartling J. Bruder J. Kunze S. Neuhoff N. (2014). Cognitive mechanisms underlying reading and spelling development in five European orthographies. Learn. Instr. 29, 65–77. 10.1016/j.learninstruc.2013.09.003

[B77] Muter V. Hulme C. Snowling M. J. Stevenson J. (2004). Phonemes, rimes, vocabulary, and grammatical skills as foundations of early reading development: evidence from a longitudinal study. Dev. Psychol. 40, 665–681. 10.1037/0012-1649.40.5.665 15355157

[B78] Nagy W. Berninger V. W. Abbott R. D. (2006). Contributions of morphology beyond phonology to literacy outcomes of upper elementary and middle-school students. J. Educ. Psychol. 98, 134–147. 10.1037/0022-0663.98.1.134

[B79] Nagy W. Berninger V. Abbott R. Vaughan K. Vermeulen K. (2003). Relationship of morphology and other language skills to literacy skills in at-risk second grade readers and at-risk fourth grade writers. J. Educ. Psychol. 95, 730–742. 10.1037/0022-0663.95.4.730

[B80] Nation K. (2008). Learning to read words. Q. J. Exp. Psychol. 61, 1121–1133. 10.1080/17470210802034603 18609391

[B81] Nation K. Snowling M. (2004). Beyond phonological skills: broader language skills contribute to the development of reading. J. Res. Read. 27, 342–356. 10.1111/j.1467-9817.2004.00238.x

[B82] O'Connor M. Arnott W. McIntosh B. Dodd B. (2009). Phonological awareness and language intervention in preschoolers from low socio-economic backgrounds: a longitudinal investigation. Br. J. Dev. Psychol. 27, 767–782. 10.1348/026151008X372492 19994478

[B83] Ouellette G. Beers A. (2010). A not-so-simple view of reading: how oral vocabulary and visual-word recognition complicate the story. Read. Writ. 23, 189–208. 10.1007/s11145-008-9159-1

[B84] Ouellette G. Haley A. (2013). One complicated extended family: the influence of alphabetic knowledge and vocabulary on phonemic awareness. J. Res. Read. 36, 29–41. 10.1111/j.1467-9817.2010.01486.x

[B85] Ramirez G. Walton P. Roberts W. (2013). Morphological awareness and vocabulary development among kindergarteners with different ability levels. J. Learn. Disabil. 47, 54–64. 10.1177/0022219413509970 24306459

[B86] Raven J. C. Raven J. E. Court J. H. (1998). Manual for Raven's Progressive Matrices and Vocabulary Scales. Oxford: Oxford Psychologists Press.

[B87] Rispens J. E. McBride-Chang C. Reitsma P. (2008). Morphological awareness and early and advanced word recognition and spelling in Dutch. Read. Writ. 21, 587–607. 10.1007/s11145-007-9077-7

[B88] Roman A. A. Kirby J. R. Parrila R. K. Wade-Woolley L. Deacon S. H. (2009). Toward a comprehensive view of the skills involved in word reading in Grades 4, 6, and 8. J. Exp. Child Psychol. 102, 96–113. 10.1016/j.jecp.2008.01.004 18329037

[B89] Rosa G. J. Valente B. D. de los Campos G. Wu X. L. Gianola D. Silva M. A. (2011). Inferring causal phenotype networks using structural equation models. Genet. Sel. Evol. 43:6. 10.1186/1297-9686-43-6 21310061 PMC3056759

[B90] Sanchez M. Magnan A. Ecalle J. (2012). Knowledge about word structure in beginning readers: what specific links are there with word reading and spelling? Eur. J. Psychol. Educ. 27, 299–317. 10.1007/s10212-011-0071-8

[B91] Scarborough H. S. (2001). Connecting early language and literacy to later reading (dis)abilities: evidence, theory, and practice, in Handbook of Early Literacy Research, eds Neuman S. B. Dickinson D. K. (New York, NY: Guilford Press), 97–110.

[B92] Seymour P. H. Aro M. Erskine J. M. (2003). Foundation literacy acquisition in European orthographies. Br. J. Psychol. 94, 143–174. 10.1348/000712603321661859 12803812

[B93] Share D. (1995). Phonological recoding and self-teaching: sine qua non of reading acquisition. Cognition 55, 151–218. 10.1016/0010-0277(94)00645-2 7789090

[B94] Share D. (1999). Phonological recoding and orthographic learning: a direct test of the self-teaching hypothesis. J. Exp. Child Psychol. 72, 95–129. 10.1006/jecp.1998.2481 9927525

[B95] Share D. L. Jorm A. E. MacLean R. Matthews R. (1984). Sources of individual differences in reading acquisition. J. Educ. Psychol. 76, 1309–1132. 10.1037/0022-0663.76.6.1309

[B96] Sorbom D. (1986). Model modification. Psychometrika 54, 371–384. 10.1007/BF02294623

[B97] Sparks E. Deacon H. (2015). Morphological awareness and vocabulary acquisition: a longitudinal examination of their relationship in English-speaking children. J. Appl. Psycholinguistics 36, 299–321. 10.1017/S0142716413000246

[B98] Spencer M. Muse A. Wagner R. K. Foorman B. Petscher Y. Schatschneider C.. (2015). Examining the underlying dimensions of morphological awareness and vocabulary knowledge. Read. Writ. 28, 959–988. 10.1007/s11145-015-9557-0 26273128 PMC4530804

[B99] Spirtes P. Glymour C. Scheines R. (2001). Causation, Prediction, and Search, 2nd Edn. Cambridge: MIT Press.

[B100] Sprenger-Charolles L. Colé P. (2013). Lecture et Dyslexie: Approche Cognitive, 2nd Édn. Paris: Dunod.

[B101] Steiger J. H. (1990). Structural model evaluation and modification: an interval estimation approach. Multivar. Behav. Res. 25, 173–180. 10.1207/s15327906mbr2502_4 26794479

[B102] Suggate S. P. (2016). A meta-analysis of the long-term effects of phonemic awareness, phonics, fluency, and reading comprehension interventions. J. Learn. Disabil. 49, 77–96. 10.1177/0022219414528540 24704662

[B103] Thompson P. A. Hulme C. Nash H. M. Gooch D. Hayiou-Thomas E. Snowling M. J. (2015). Developmental dyslexia: predicting individual risk. J. Child Psychol. Psychiatry Allied Discipl. 56, 976–987. 10.1111/jcpp.12412 25832320 PMC4672694

[B104] Tighe E. L. Schatschneider C. (2015). Exploring the dimensionality of morphological awareness and its relations to vocabulary knowledge in adult basic education students. Read. Res. Q. 50, 293–311. 10.1002/rrq.102

[B105] Tse L. Nicholson T. (2014). The effect of phonics-enhanced Big Book reading on the language and literacy skills of 6-year-old pupils of different reading ability attending lower SES schools. Front. Psychol. 5:1222. 10.3389/fpsyg.2014.01222 25431560 PMC4230049

[B106] Tunmer W. E. Chapman J. W. (2012). The simple view of reading redux: vocabulary knowledge and the independent components hypothesis. J. Learn. Disabil. 45, 453–466. 10.1177/0022219411432685 22293683

[B107] Tunmer W. Greaney K. (2010). Defining dyslexia. J. Learn. Disabil. 43, 229–243. 10.1177/0022219409345009 19834134

[B108] Vandenberghe R. Wang Y. Nelissen N. Vandenbulcke M. Dhollander T. Sunaert S.. (2013). The associative-semantic network for words and pictures: effective connectivity and graph analysis. Brain Lang. 127, 264–272. 10.1016/j.bandl.2012.09.005 23084460

[B109] Wagner R. K. Herrera S. K. Spencer M. Quinn J. M. (2015). Reconsidering the simple view of reading in an intriguing case of equivalent models: commentary on tunmer and chapman (2012). J. Learn. Disabil. 48, 115–119. 10.1177/0022219414544544 25095787 PMC4318790

[B110] Wagner R. K. Torgesen J. K. Laughon P. L. Simmons K. Rashhotte C. A. (1993). Development of young readers phonological processing abilities. J. Educ. Psychol. 85, 83–103. 10.1037/0022-0663.85.1.83

[B111] Walley A. C. Metsala J. L. Victoria M. Garlock V. M. (2003). Spoken vocabulary growth: its role in the development of phoneme awareness and early reading ability. Read. Writ. 16, 5–20. 10.1023/A:1021789804977

[B112] Whittaker J. (1990). Graphical Models in Applied Multivariate Statistics. Chichester: Wiley.

[B113] Wolter J. A. Wood A. D'zatko K. W. (2009). The influence of morphological awareness on the literacy development of first-grade children. Lang. Speech Hear. Serv. Sch. 40, 286–298. 10.1044/0161-1461(2009/08-0001) 19564443

[B114] Wright S. (1921). Correlation and causation. J. Agric. Res. 20, 557–585.

